# Diagnostic accuracy of antigen-detection rapid diagnostic tests for diagnosis of COVID-19 in low-and middle-income countries: A systematic review and meta-analysis

**DOI:** 10.1371/journal.pgph.0000358

**Published:** 2022-04-11

**Authors:** Sagar Pandey, Arisa Poudel, Dikshya Karki, Jeevan Thapa

**Affiliations:** 1 BP Koirala Institute of Health Sciences, Dharan, Nepal; 2 Institute of Medicine, Tribhuvan University, Kathmandu, Nepal; 3 Kathmandu Medical College, Kathmandu, Nepal; 4 Patan Academy of Health Sciences, Lalitpur, Nepal; Universidad Autonoma de Baja California, MEXICO

## Abstract

Antigen detection rapid diagnostic tests (Ag-RDTs) used for detecting severe acute respiratory syndrome coronavirus 2 (SARS-CoV-2) nucleocapsid protein are inexpensive, faster and easy to use alternative of Nucleic Acid Amplification Test (NAAT) for diagnosis of Coronavirus disease 2019 (COVID-19). In this systematic review and meta-analysis, we assessed the diagnostic accuracy of Ag-RDTs in low and middle-income countries (LMICs). We included studies that evaluated the diagnostic accuracy of Ag-RDTs (sensitivity and specificity) against reverse transcription-polymerase chain reaction (RT-PCR) as a reference standard. The study population comprised of people living in LMICs irrespective of age and gender, who had undergone testing for COVID-19. We included peer reviewed prospective or retrospective cohort studies, cross-sectional studies, case control studies, randomized clinical trials (RCTs) as well as non-randomized experimental studies which addressed the review question. A systematic search was conducted in PubMed, CINAHL, Embase, Scopus, and Google Scholar to identify studies published between 1 January, 2020 and 15 August, 2021. The Quality Assessment of Diagnostic Accuracy Studies (QUADAS)-2 tool was used to assess the methodological quality of studies. The analysis was done using Review Manager 5.4 and R software 4.0.2. From the total of 12 diagnostic accuracy studies with 4,817 study participants, pooled sensitivity and specificity were 78.2% and 99.5% respectively. Sensitivity was marginally higher in subgroup analysis based on studies with low risk of bias and applicability concerns (78.9%) and studies using SD Biosensor Ag-RDT (79.4%). However, an inverse relation between cycle threshold (Ct) and sensitivity of Ag-RDT was not seen. The review demonstrated pooled sensitivity value approaching the minimum performance requirement for diagnosis of COVID-19 by WHO with specificity value meeting the specified requirement. Ag-RDTs, therefore have the potential to be used as a screening tool for SARS-CoV-2 detection in low resource settings where RT-PCR might not be readily accessible. However, false negative results need to be interpreted with caution.

## Introduction

Coronavirus disease 2019 (COVID-19) has been spreading rapidly across the globe causing loss of millions of lives since it was first reported in Wuhan, China and later declared a pandemic on 11^th^ March, 2020 [1]. In addition to strategies like vaccination against COVID-19, contact tracing, and home isolation for potential exposure to or diagnosis of COVID-19, early diagnosis and community screening for COVID-19 are crucial to control the spread of disease [2]. CDC has outlined viral test (Nucleic Acid Amplification Test (NAAT) and antigen tests) and antibody tests as methods for testing current and past infection with COVID-19 respectively [3]. Although, NAAT is the preferred initial diagnostic test for COVID-19, its use in low resource settings is limited by its cost and need for qualified clinical laboratory personnel [4]. Antigen detection rapid diagnostic tests (Ag-RDTs) used for detecting SARS-CoV-2 nucleocapsid protein are inexpensive, faster and easy to use alternative of NAAT for COVID-19 testing. This trade-off for the ease-of-use of Ag-RDTs is decrease in sensitivity and specificity of test [2].

Various studies are done to evaluate the diagnostic accuracy of Ag-RDTs that vary in study design, study setting and study population along with various brands being evaluated. In a review evaluating the diagnostic accuracy of rapid point-of care antigen test for COVID-19 with studies mainly from Europe and North America, summary sensitivities were from 34.1% and 88.1% for Coris Bioconcept and SD Biosensor STANDARD Q Ag-RDTs respectively whereas overall summary specificity was 99.6% [5]. However, such study assessing the diagnostic accuracy of Ag-RDTs for study population belonging to low and middle income countries (LMIC) has not been done yet. We conducted this review to evaluate the diagnostic accuracy of Ag-RDTs, with RT-PCR as a reference standard, with study population from low and middle income countries which differ not only with the type of Ag-RDTs used but also with the testing conditions, compliance with the manufactures instruction for use, adherence to guidelines, specified method of collection and handling of specimens, etc.

## Methods

### Protocol and registration

We developed, conducted and reported this review following Preferred reporting items for systematic review and meta-analysis of diagnostic test accuracy studies (PRISMA-DTA) [6]. PRISMA-DTA checklist is available in S1 File. The protocol was drafted and registered before conducting the systematic review and meta-analysis in international prospective register for systematic review (PROSPERO) with registration number: CRD42021259260 which is available in S2 File.

### Eligibility criteria

The eligibility criteria for studies was formulated based on population, intervention, comparator and outcome (PICO) for the review question. We included studies that evaluated the diagnostic accuracy of Ag-RDTs in terms of sensitivity and specificity, against RT-PCR as a reference standard. The study population comprised of people living in LMICs, who had undergone testing for COVID-19. Only original studies in English language published in the year 2020 and up until August 15, 2021 that described their methods and reported enough data for the construction of the standard two-by-two table were included.

We included peer reviewed prospective or retrospective cohort studies, cross-sectional studies, case control studies, randomized clinical trials as well as non-randomized experimental studies which addressed the review question. Editorials, letters to the editors, and conference abstracts were excluded since they contained insufficient information required for conducting a review. Studies in which diagnostic accuracy of Ag-RDT was determined in a sample of less than 100 was excluded so that a more reliable estimate of diagnostic accuracy could be obtained.

### Index test

Ag-RDTs are designed to directly detect SARS CoV-2 viral proteins produced by the replicating virus. Most Ag-RDTs for COVID-19 use a sandwich immunodetection method employing a simple-to-use lateral flow test format with techniques such as enzyme-linked immunosorbent assay (ELISA), chromogenic based or fluorescence-based detection. In the case of SARS-CoV-2, Ag-RDTs the target analyte is often the virus’ nucleocapsid protein. After collecting the respiratory specimen and applying it to the test strip, results are read by the operator within 10 to 30 minutes with or without the aid of a reader instrument. Most of the currently manufactured tests require nasal or nasopharyngeal or oropharyngeal swab samples or bronchoalveolar lavage/ endotracheal aspirate [7, 8]. The diagnostic test accuracy of Ag-RDTs was measured in terms of sensitivity and specificity of the test to detect a SARS CoV-2 infection compared with a reference standard, RT-PCR.

Sensitivity is the percentage of cases positive by NAAT reference standard that are detected as positive by the Ag-RDTs under evaluation. Specificity is the percentage of cases negative by a NAAT reference standard that are detected as negative by the Ag-RDTs under evaluation [9].

### Reference standard

Reverse transcription polymerase chain reaction (RT-PCR) for COVID-19 is a molecular test that detects the genetic material (RNA) of SARS-CoV-2 responsible for coronavirus disease. It combines the laboratory technique of reverse transcription and polymerase chain reaction which amplifies specific complementary DNA (cDNA) targets [10]. Typically samples collected from nasopharyngeal, oropharyngeal or anterior nasal swab is used. The number of amplification cycles required to reach the level of detection is reported as cycle threshold (Ct) value of RT-PCR. The fewer the Ct value, the higher the viral RNA load [4]. Among the currently available diagnostics tests for SARS-CoV-2, RT-PCR is considered a gold standard due to its improved sensitivity and specificity compared to serological methods of virus detection [11].

### Search strategy

We conducted a systematic search for relevant articles in the following databases: PubMed, CINAHL, Embase, Scopus, and Google Scholar. The search strategy included keywords like "coronavirus infections", "COVID-19”, "coronavirus disease 2019", "COVID19" "SARS-CoV-2", "covid-19 testing", "Antigen based rapid diagnostic test", "diagnostic test", "lateral flow antigen", "lateral flow antigen detection", "lateral flow assay", "Point of care testing" and a list of all LMICs as listed by the World Bank in 2020 with a combination of Boolean operators [12]. We used filters such as studies published between the year 2020 and 2021 to limit the number of irrelevant articles. Furthermore, we also conducted a free hand search for relevant articles in the references section of articles included in the review to avoid missing the eligible studies. We included the studies published upto August 15, 2021. The full search strategy used in PubMed is available in S3 File.

### Data screening and extraction

Our review team comprised of four members to search, extract and analyze the available data, perform risk of bias assessment and synthesis of results. Three reviewers SP, AP and DK independently screened and retrieved the studies using the search strategy and from additional sources. Studies were further reviewed for eligibility by screening titles followed by abstracts. Full-text articles of narrowed down abstracts were then be assessed for eligibility. SP and JT rechecked the decisions and upon disagreements, all four reviewers reviewed the inclusion and exclusion criteria and decision for inclusion was made based on majority’s decision. SP made the final decision after thorough reviewing when an agreement could not be reached.

Zotero, a research tool to collect, organize, and manage research publications was used to keep a record, and remove duplicates. All studies retrieved from our search strategies matching our PICO questions and inclusion criteria were imported to Zotero. Titles of the studies was first screened followed by abstracts. Screened studies were placed into appropriate subfolders created in Zotero based on decisions to include or exclude. Full text of all eligible studies were retrieved and assessed for eligibility. Final data was extracted from the included studies into an excel spreadsheet. The following information was extracted (if available): demographics details of study participants, total number of study participants, type of study, type of specimen used in Ag-RDTs and outcome measures recorded i.e. sensitivity and specificity. In case of any missing data, authors of the study were contacted for additional information. SP and AP independently extracted the data, and DK and JT checked the extracted data. Upon disagreements, all four reviewers reviewed the final extracted data, and disagreements were resolved through discussion on inclusion and exclusion criteria via majority’s decision.

### Risk of bias

The Quality Assessment of Diagnostic Accuracy Studies (QUADAS)-2 tool was used to assess the methodological quality of all studies to be included in this systematic review [13]. QUADAS-2 consists of four key domains: patient selection, index test, reference standard, and flow and timing. We assessed all the domains for risk of bias potential using different signaling questions, and the first three domains for applicability concerns. Based on this, risk of bias was judged as “low,” “high,” or “unclear”. Summary of results of QUADAS-2 for all included studies is presented in a tabular and graphical displays generated via Review manager 5.4. Two reviewers SP and JT independently assessed the risk of bias and the quality of the study. Disagreements between the authors were resolved by further discussion and consensus with other researchers.

### Statistical analysis and data synthesis

We extracted data from the studies to construct standard two-by-two table used to calculate sensitivity and specificity. The studies were grouped based on a) clinical presentation at the time of testing i.e. symptomatic, asymptomatic or mixed, b) type of commercial Ag-RDT used i.e. SD Biosensor or tests other than SD Biosensor, c) Ct cut-off value for positive RT-PCR i.e. ≤40, <40, <35, <32 and non-specified, d) risk of bias and concerns regarding applicability for patient selection i.e. low or high, and e) concordance of samples used for index test and reference standard. For subgroup based on commercial Ag-RDT used, tests used in more than two studies were made a separate category (e.g. SD Biosensor) whereas those used in only one study were lumped into a common category of tests other than SD Biosensor. Likewise, subgroup analysis based on Ct cut-off value for positive RT-PCR was done only for studies where the cut-off value used in the study was clearly mentioned. A random effects model with 95% confidence interval (CI) was used to calculate pooled sensitivity and specificity using Review Manager (Revman) 5.4. Higgins’ I square was used to measure heterogeneity (I^2^ > 50% indicating substantial heterogeneity) [14]. Subgroup analyses were conducted in R software version 4.0.2 using R package “mada” version 0.5.8 and were predefined based on: clinical presentation at the time of testing, type of commercial Ag-RDT used, Ct cut off value for positive RT-PCR, risk of bias and concerns regarding applicability for patient selection, and concordance of samples used for index test and reference standard. Bivariate analysis was done using “reitsma” command from R package “mada” to obtain Hierarchical Summary Receiver-Operating Characteristics (HSROC) parameters, which was then used in Revman to obtain HSROC curve.

## Results

### Study selection and characteristics of included studies

**Fig 1:**
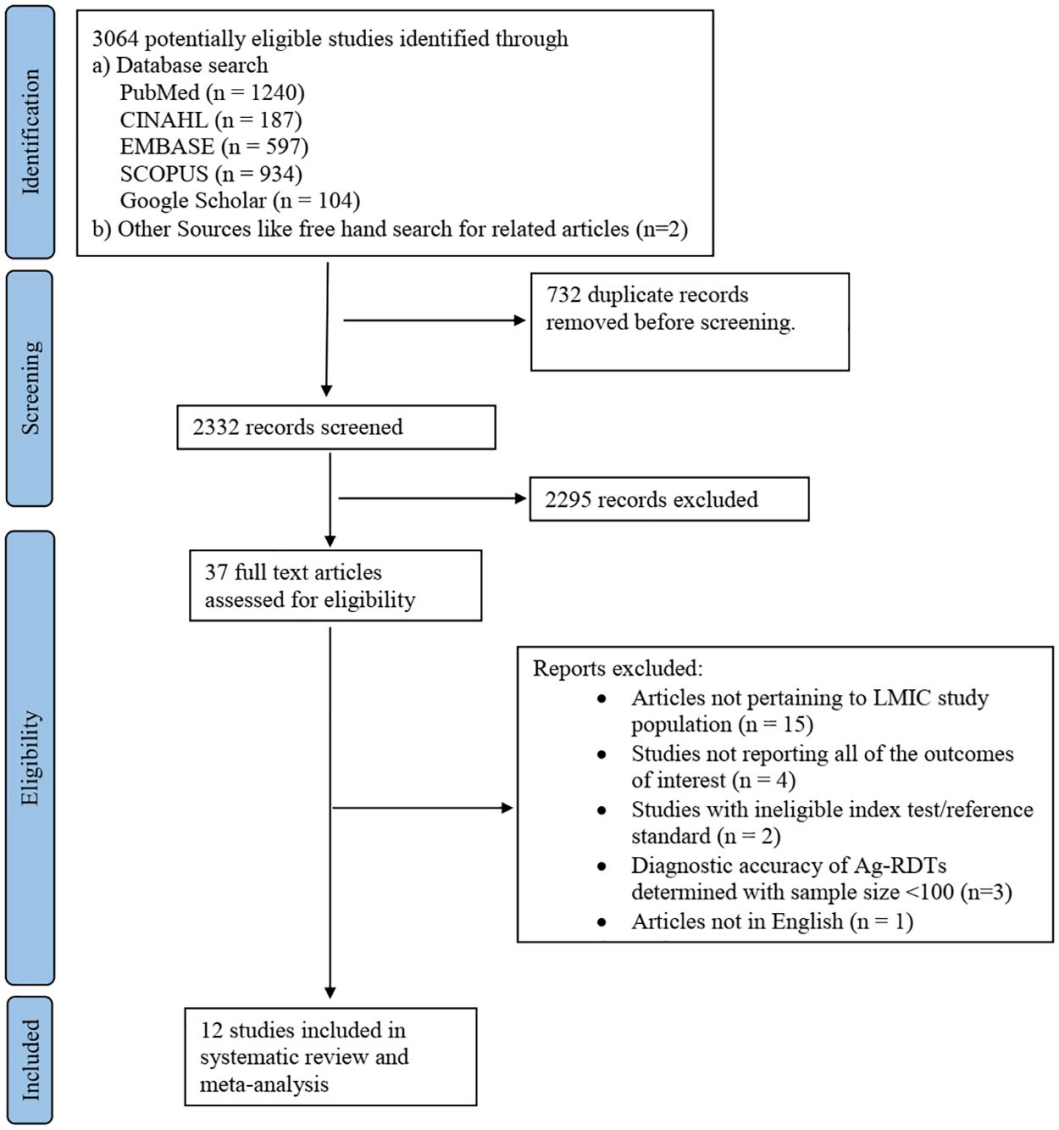
PRISMA 2020 flow diagram.

We identified 3,064 records after performing a systematic search for articles. After removing 732 duplicate records, 2,332 studies underwent title and abstract screening. A total of 2295 studies did not address the review question and were therefore, excluded. Full text screening of remaining 37 studies yielded 12 studies which met the eligibility criteria and were included in the review. Out of the 25 excluded articles, 15 did not involve LMIC study population, four did not report all of the outcomes of interest, two had ineligible index test/reference standard, three had evaluated the diagnostic accuracy of Ag-RDT on a sample of less than 100 and one was not in English. PRISMA flow diagram for selection of studies is depicted in Fig 1.

The 12 studies included diagnostic accuracy studies were conducted in Cameroon, China, India, Mexico, Nepal, Serbia, Thailand, and Uganda which enrolled a total of 4,817 study participants. Out of the total 12 studies, 12 of them used consecutive or random sampling method as a method of patient enrollment whereas three of them used non-random method i.e. Pena-Rodriguez et al. [15]. used known cases of patients with positive RT-PCR for COVID-19 as one of the inclusion criteria, Nalumansi et al. [16] used low risk volunteer as non-case controls whereas Shrestha et al. [17] used convenience sampling method. Symptomatic patients suspected of having COVID-19 were the study population in two studies whereas another two studies used asymptomatic patients as the study population. A combination of symptomatic and asymptomatic patients were used in rest of the eight studies. Standard Q COVID-19 Ag test (SD Biosensor) was the Ag-RDT test used in majority of the studies (seven out of 12) whereas the rest of the studies used RapiGEN—BIOCREDIT COVID-19 Ag test, Coris bioconcept COVID-19 Ag respi-strip test, PathoCatch/ACCUCARE diagnostic assay, etc. Nasopharyngeal swab was used as a sample for Ag-RDT in most of the studies whereas two of the studies used nasal swab and one used a combination of nasopharyngeal and throat swab, endotracheal aspirate and sputum sample. Ct reference standard used for RT-PCR was specified in six out of twelve studies whereas the rest of the studies did not mentioned the Ct value. Table 1 summarizes the study characteristics of the included studies.

**Table 1:** Characteristics of included studies.

Study	Study Design	Study Population (age distribution)	Study Location	Sample type	Commercial name of Ag-RDT used	Clinical presentation at the time of testing	Sample size (Male/Female)
Diao, 2021 [18]	Prospective study	Adults (16–75 years)	China	NPS	Not available	Symptomatic	251 (122/129)
Agrawal, 2021 [19]	Prospective study	Adults and Children (2–85 years)	India	NS	Standard Q COVID-19 Ag test (SD Biosensor)	Symptomatic and Asymptomatic	467 (239/228)
Kim, 2021 [20]	Prospective & retrospective study	Not Specified	India	NPS	GenbodyCOVID-19 Ag test COVAG025	Symptomatic and Asymptomatic	200 (not specified)
Nalumansi,2020 [16]	Prospective cross-sectional study	Adults (31–39 years)	Uganda	NS	Standard Q COVID-19 Ag test (SD Biosensor)	Symptomatic and Asymptomatic	262 (234/28)
Boum, 2021 [21]	Prospective study	Adults (at least 21 years)	Cameroon	NPS	SD Biosensor test	Symptomatic and Asymptomatic	1090(not specified)
Pena-Rodriguez, 2021 [15]	Prospective cross-sectional study	Not Specified, Mean age: 36.6±13.16	Mexico	NPS/OPS	Standard Q COVID-19 Ag test (SD Biosensor)	Symptomatic and Asymptomatic	369 (154/215)
Gupta, 2021 [22]	Prospective cross-sectional study	Adults (>18 years)	India	NPS	Standard Q COVID-19 Ag test (SD Biosensor)	Symptomatic and Asymptomatic	330 (231/99)
Chaimayo, 2020 [23]	Prospective study	Adults (21–72 years)	Thailand	NPS and TS, ETA, Sp.	Standard Q COVID-19 Ag test (SD Biosensor)	Symptomatic and Asymptomatic	454(not specified)
Shrestha, 2020 [17]	Prospective observational study	Adults and Children (13–72 years)	Nepal	NPS	RapiGEN—BIOCREDIT COVID-19 Ag	Asymptomatic	113 (24/89)
Ristic, 2021 [24]	Prospective study	Adults and Children (14–91 years)	Serbia	NPS	Standard Q COVID-19 Ag test (SD Biosensor)	Symptomatic	120 (63/57)
Thakur, 2021 [25]	Prospective cross- sectional study	Adults (18–89 years)	India	NPS and OPS	PathoCatch/ACCUCARE	Asymptomatic	677 (372/305)
Kanaujia, 2021 [26]	Prospective cross-sectional study	Adults and Children (13–90 years)	India	NPS	Coris bioconcept COVID-19 ag respi-strip test	Symptomatic and Asymptomatic	484 (261/223)

NPS = Nasopharyngeal Swab, NS = Nasal Swab, OPS = Oropharyngeal Swab, TS = Throat Swab, ETA = Endotracheal Aspirate, Sp. = Sputum.

### Methodological quality of studies

Risk of bias on selection of patients was considered low in nine out of 12 included studies as they avoided case-control design, inappropriate exclusions of patients and non-random sampling of patients. Similarly, the included patients matched the review question in all of those studies. In contrast, the remaining three studies had high risk of bias on selection of patients along with high concerns regarding applicability as they used non-random method of patient sampling.

With regard to index test, its conduct or interpretation was considered to have low risk of bias in six studies i.e. index test was interpreted without knowledge of the reference standard and a pre-specified threshold was used. However, in remaining five studies sufficient information to clearly judge the risk of bias was not provided. In one study, index test was interpreted with knowledge of the reference standard leading to high risk of bias. Index test, its conduct or interpretation matched the review question in eight studies whereas it was unclear in remaining four studies as sufficient information was not provided regarding whether trained personnel was involved in sample collection and test administration.

Since the estimate of test accuracy is made on the assumption that the reference standard is 100% sensitive, RT-PCR alone as a reference standard, its conduct or interpretation was considered to have high risk of bias in all of the included studies as the test lacked the sensitivity of 100%. In contrast, concern regarding applicability was low for all studies except one which used real time micro PCR analyzer as a reference standard.

**Fig 2:**
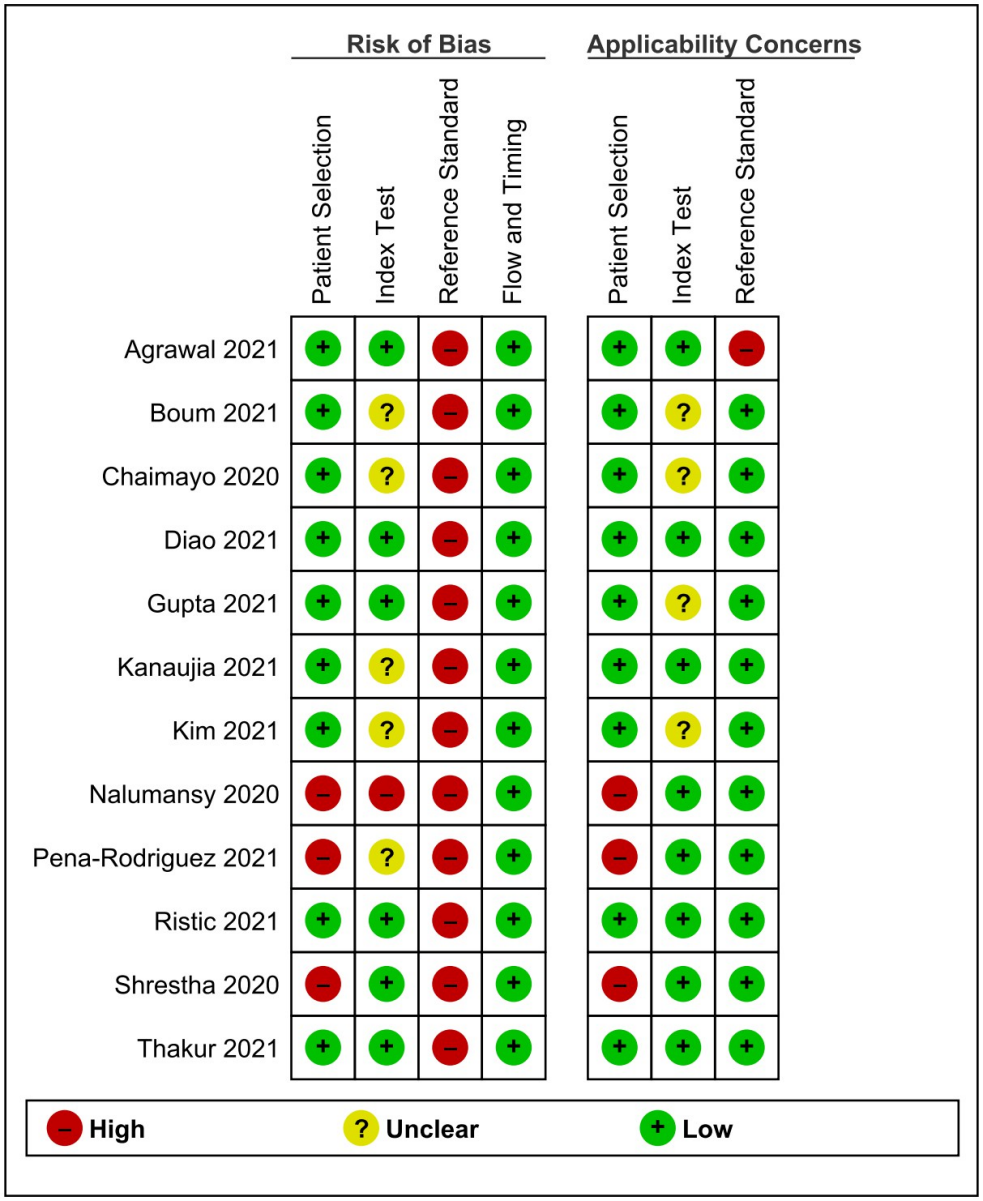
Summary of risk of bias and applicability concerns for each included studies for each separate domain.

**Fig 3:**
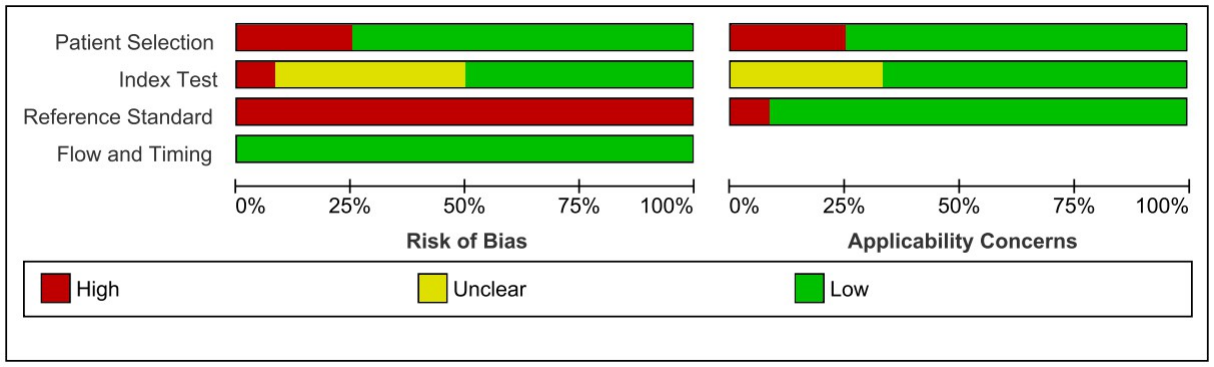
Summary of risk of bias and applicability concerns across the included studies.

Under flow and timing domain, index test(s) and reference standard were collected at the same time in all of the studies. Similarly, all of the patients received the same reference standard and also were included in the analysis leading to low risk of bias with regard to patient flow. Methodological quality of included studies is presented in Figs 2 and 3.

### Analysis of specific tests

Table 2 summarizes the sensitivity, specificity, positive likelihood ratio (LR+), negative likelihood ratio (LR-), diagnostic odds ratio (DOR) along with true positive(TP), false positive(FP), true negative(TN) and false negative(FN) values for each of the Ag-RDTs used in included studies.

**Table 2:** Summary diagnostic accuracy data for included studies.

SN	Study	TP	FP	TN	FN	Sensitivity	Specificity	LR(+)	LR(-)	DOR
1	Diao,2021	152	0	50	49	0.756	1.000	-	0.244	-
2	Agrawal,2021	26	2	436	3	0.897	0.995	196.345	0.104	1889.333
3	Kim,2021	94	0	100	6	0.940	1.000	-	0.060	-
4	Nalumansi,2020	63	13	159	27	0.700	0.924	9.262	0.325	28.538
5	Boum,2021	170	54	745	121	0.584	0.932	8.644	0.446	19.383
6	Pena-Rodriguez,2021	79	0	265	25	0.760	1.000	-	0.240	-
7	Gupta,2021	63	1	252	14	0.818	0.996	207.000	0.183	1134.000
8	Chaimayo,2020	59	5	389	1	0.983	0.987	77.487	0.017	4590.200
9	Shrestha,2020	40	0	66	7	0.851	1.000	-	0.149	-
10	Ristic,2021	25	0	77	18	0.581	1.000	-	0.419	-
11	Thakur,2021	29	1	592	55	0.345	0.998	204.726	0.656	312.145
12	Kanaujia,2021	136	2	293	53	0.720	0.993	106.138	0.282	375.925

TP = True Positive, FP = False Positive, TN = True Negative, FN = False Negative, LR(+) = Positive Likelihood Ratio, LR(-) = Negative Likelihood Ratio.

**Fig 4:**
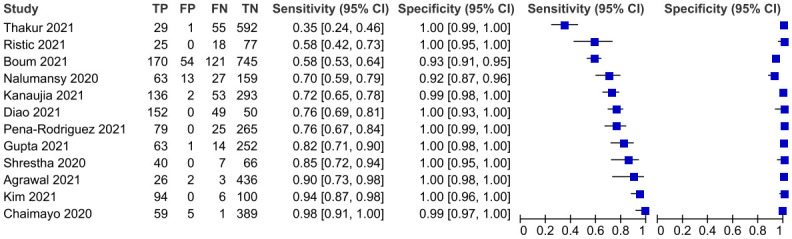
Coupled forest plot of sensitivity and specificity.

A coupled forest plot of sensitivity and specificity with an ascending display of sensitivity values is shown in Fig 4 to evaluate threshold effect as a source of heterogeneity for diagnostic test accuracy study. On increasing the sensitivity values, specificity remained more or less constant thus negating the possibility of a threshold effect.

**Fig 5:**
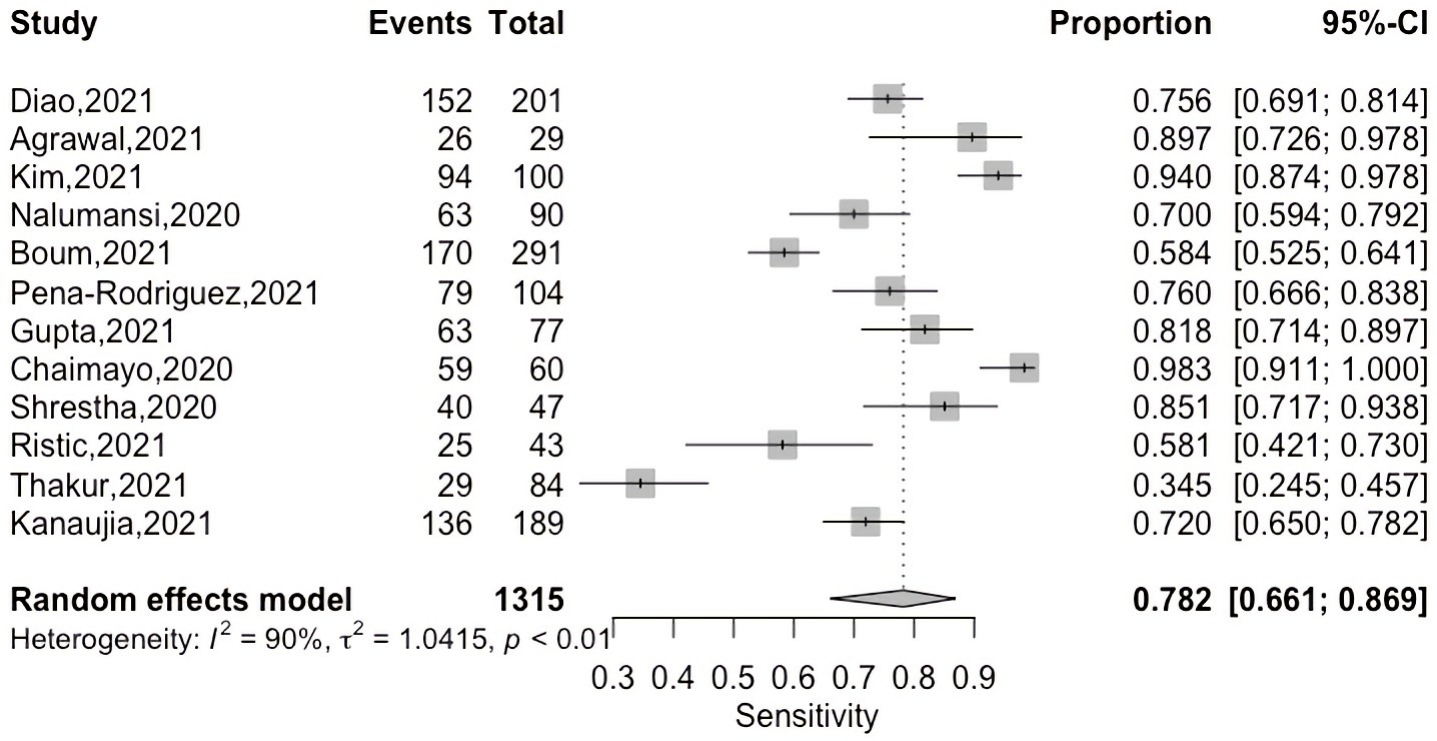
Pooled sensitivity of included studies.

**Fig 6:**
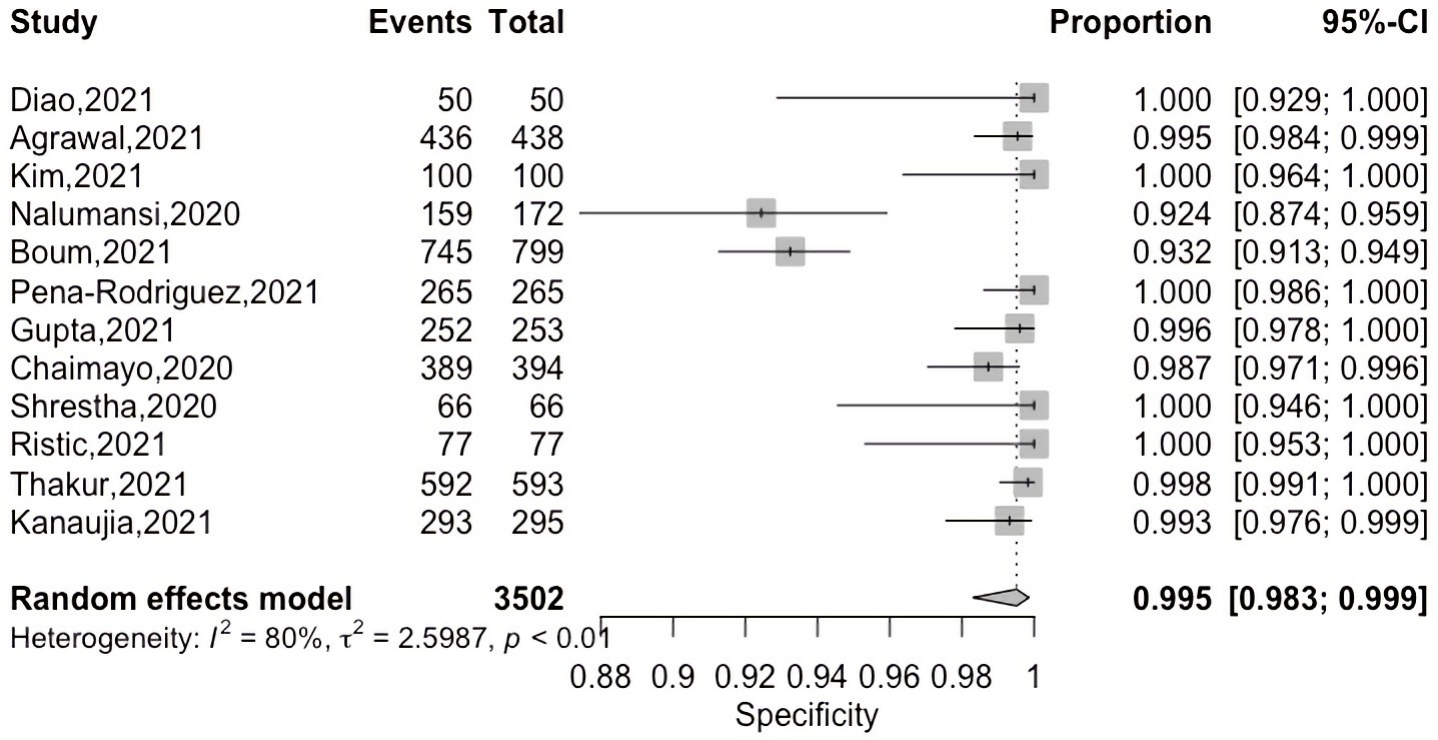
Pooled specificity of included studies.

The overall pooled sensitivity and specificity of 12 Ag-RDTs was 78.2 (66.1–86.9, 95% CI) and 99.5 (98.3–99.9, 95% CI) respectively as demonstrated on Figs 5 and 6 below.

On subgroup analysis, pooled specificity was consistently higher than sensitivity and above 99% except for subgroup defined by Ct cutoff value used for positive RT-PCR (i.e. for Ct <40: 96.9% with 95% CI: 89.3–99.1%). Pooled sensitivity for subgroup analysis based on commercial type of Ag-RDT used was higher for the SD Biosensor test (i.e. 79.4% with 95% CI: 64.2–89.3%) compared to that for tests other than SD Biosensor (i.e.76.3% with 95% CI: 54.7–89.6%). Furthermore, subgroup defined by the clinical presentation at the time of testing had sensitivity ranging from 83.2% (for tests performed on either symptomatic or asymptomatic patients) to 62.9% (for tests performed on only asymptomatic patients). In addition, pooled sensitivity for subgroup based on Ct cut off value for positive RT-PCR was highest when threshold of <40 was used (91.5%) and lowest for threshold of <32 (34.5%). On the contrary, pooled sensitivity for Ct threshold of ≤40 was 69.6%. Surprisingly, pooled sensitivity values for subgroup based on sample concordance for index test and reference standard was higher for studies using different samples (81.1%) than that for those with same sample (77.7%). Pooled sensitivity was higher for tests used in studies with low risk of bias and low concerns regarding applicability for patient selection (78.9%) when compared to that for tests used in studies with high risk of bias and concerns regarding applicability (75.5%). Each of the subgroups estimating sensitivity had higher level of heterogeneity except for the subgroup defined by the risk of bias and concern regarding applicability for patient selection (i.e.I^2^ = 46% for high risk of bias and high concern regarding applicability). Tables 3 and 4 demonstrates the pooled sensitivity and specificity of Ag-RDTs for detection of SARS-CoV-2 on subgroup analysis.

**Table 3:** Pooled sensitivity of Ag-RDTs for detection of SARS-CoV-2 on subgroup analysis.

Subgroups		Pooled Sensitivity (95% CI)	Number of studies analyzed	Total number of events	Heterogeneity	
					I^2^	p-Value
**Based on clinical presentation at the time of testing**	**Symptomatic**	69.6 (56.6–80.1)	2	244	81	0.02
	**Asymptomatic**	62.9 (23.8–90.2)	2	131	96	<0.01
	**Symptomatic/ Asymptomatic**	83.2 (70.8–91.0)	8	940	88	<0.01
**Based on type of Commercial Ag-RDT used**	**SD Biosensor test**	79.4 (64.2–89.3)	7	694	94	<0.01
	**Other**	76.3 (54.7–89.6)	5	621	85	<0.01
**Based on Ct cut off value for positive RT-PCR**	**≤40**	69.6 (56.6–80.1)	2	244	81	0.02
	**<40**	91.5 (49.1–99.2)	2	150	90	<0.01
	**<35**	76.0 (66.8–83.2)	1	104	NA	NA
	**<32**	34.5 (25.2–45.3)	1	84	NA	NA
	**Not specified**	82.0 (69.9–89.9)	6	733	91	<0.01
**Based on risk of bias and concerns regarding applicability for patient selection**	**Low**	78.9 (62.0–89.6)	9	1074	92	<0.01
	**High**	75.5 (68.9–81.1)	3	241	46	0.16
**Based on concordance of samples used for index test and reference standard**	**Yes**	77.7 (68.2–85.0)	9	1067	86	<0.01
	**No**	81.1 (31.6–97.5)	3	248	96	<0.01

**Table 4:** Pooled specificity of Ag-RDTs for detection of SARS-CoV-2 on subgroup analysis.

Subgroups		Pooled Specificity (95% CI)	Number of studies analyzed	Total number of events	Heterogeneity	
					I^2^	p-Value
**Based on clinical presentation at the time of testing**	**Symptomatic**	100 (0–100)	2	127	0	1
	**Asymptomatic**	99.8 (98.9–100)	2	659	0	1
	**Symptomatic/ Asymptomatic**	99.1 (97.1–99.8)	8	2716	84	<0.01
**Based on type of Commercial Ag-RDT used**	**SD Biosensor test**	99.1 (96.3–96.8)	7	2398	83	<0.0001
	**Other**	99.7 (99.2–99.9)	5	1104	0	<0.01
**Based on Ct cut off value for positive RT-PCR**	**≤40**	100 (0–100)	2	127	0	1
	**<40**	96.9 (89.3–99.1)	2	566	92	<0.01
	**<35**	100 (0–100)	1	265	NA	NA
	**<32**	99.8 (98.8–100)	1	593	NA	NA
	**Not specified**	99.4 (97.4–99.9)	6	1951	84	<0.01
**Based on risk of bias and concerns regarding applicability for patient selection**	**Low**	99.5 (98.3–99.8)	9	2999	85	<0.01
	**High**	99.8 (62.4–100.0)	3	503	0	1
**Based on concordance of samples used for index test and reference standard**	**Yes**	99.4 (97.2–99.9)	9	2250	76	<0.01
	**No**	99.7 (98.2–99.9)	3	1252	42	0.18

### HSROC curve

**Fig 7:**
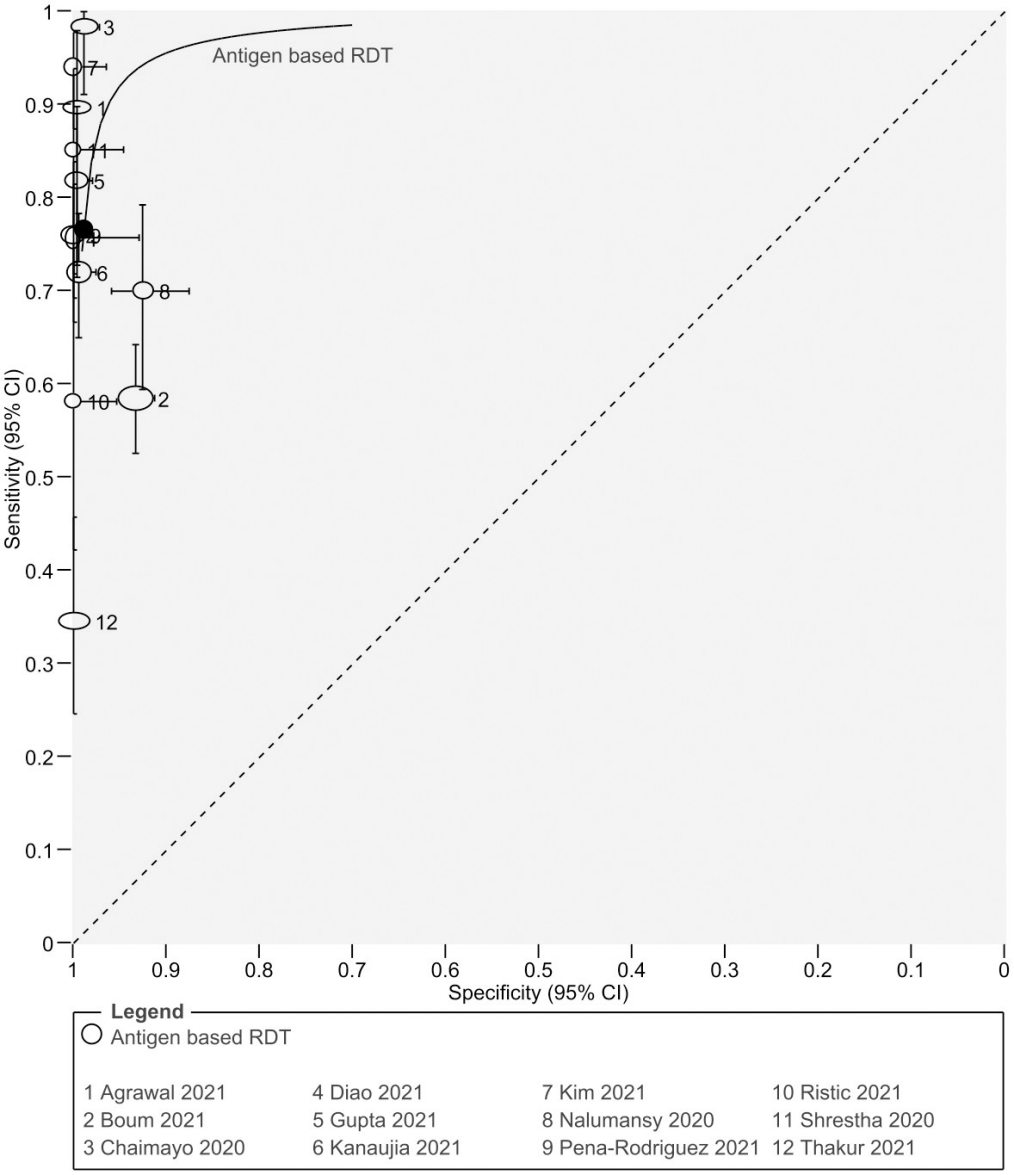
Hierarchical Summary Receiver-Operating Characteristics (HSROC) curve of the studies.

HSROC parameters were obtained from bivariate analysis using the reitsma function in mada package of R. Then the parameter values were added to the Revman to generate the HSROC curve. Area under the curve (AUC) was found to be 0.972. The HSROC curve is shown in Fig 7.

## Discussion

This systematic review and meta-analysis on 12 studies measured the diagnostic accuracy of Ag-RDTs for study population limited to LMICs. Pooled sensitivity from all of the studies (78.2%) was lower than the minimum performance requirement specified by WHO (≥80%) whereas specificity was well above the cut-off of ≥97% (99.5%) [2]. This could be attributed to factors like study design and characteristics of participants, specimen used for Ag-RDT and RT-PCR, quality and commercial type of Ag-RDT, etc. Similar factors were highlighted in WHO interim guidance on antigen detection using rapid immunoassay for selecting Ag-RDTs for enhanced diagnostic accuracy like selection of patients, study sites, manufacturing quality and storage conditions, and clarity of instructions for use, etc [2]. In addition, diagnostic accuracy of Ag-RDT was higher among symptomatic patients than asymptomatic patients, similar to the findings of systematic reviews by Brummer et al. and Khandker et al. [27, 28]. Furthermore, higher diagnostic accuracy among symptomatic patients is further supported by the findings of a study by Kociolek et al. where asymptomatic patients were found to have a lower viral load, which is a predictor of sensitivity, than symptomatic patients [29]. Although studies have reported an inverse correlation between sensitivity of Ag-RDTs and Ct value, such finding was not observed in this review [28].

This review was conducted to bridge the research gap with regard to diagnostic accuracy of Ag-RDTs for diagnosis of SARS-CoV-2 in LMIC. Khandker et al. in their review of diagnostic accuracy of rapid antigen test kits reported the sensitivity of rapid antigen test to be higher in population of Europe and America as compared to that in Asia and Africa i.e. low resource settings. The finding was attributed to the repetitive freeze-thaw process during transportation owing to lack of in-situ manufacture of kits in Asia and Africa in most instances [28]. However, the diagnostic accuracy of SD Biosensor Ag-RDT, the most commonly used test among included studies in the review, was fairly high (Sensitivity:79.4%, Specificity:99.1%) and approaching the minimum WHO performance requirement [2].

The main strength of this study lies in its comprehensive approach to review the available evidence on diagnostic accuracy of Ag-RDTs and provide some of the earliest reported evidence on their diagnostic accuracy with a study population narrowed to people living in LMIC. Furthermore, we only included peer reviewed published articles in the review to avoid skewing of the results by the studies from non-peer reviewed studies. Similarly, we followed rigorous methods for accurate risk of bias assessment along with screening for articles, study selection and data extraction.

The main limitations of this review is the methodological quality of the included studies. Three of the twelve studies used non-random method of patient selection whereas Ct threshold for RT-PCR was specified in only six out of 12 studies. Since, Ct threshold is an indirect measure of viral load of the specimen, assessing the diagnostic accuracy without its knowledge limits the comparability of the studies [30]. Furthermore, the risk of bias for the domain reference standard was high in all of the included studies as RT-PCR itself does not has an accuracy of 100% and a more comprehensive assessment with a combination of parameters should be set as a reference standard to access the diagnostic accuracy of Ag-RDTs. Interpretation of findings of subgroup analysis must be done taking into account the higher level of heterogeneity for all of the subgroups except that for the one based on risk of bias and concern regarding applicability for patient selection with low level of heterogeneity.

Despite the limitations of the study, this review provides a fresh and a unique perspective on diagnostic accuracy of Ag-RDTs in LMICs which has a significant public health implication in regards to the widespread use of Ag-RDTs over RT-PCR for COVID-19 testing. Diagnostic accuracy of Ag-RDTs approaching the near recommended performance requirement specified by WHO provides an evidence based rationale behind the use of Ag-RDT to address the diagnostic challenge for COVID-19 in LMICs. However, further studies to evaluate diagnostic accuracy of Ag-RDT using reference standard with near 100% diagnostic accuracy, achieved via combination of clinical, imaging and laboratory tests, to measure a true disease status, should be conducted to measure the true test accuracy. In addition, diagnostic test accuracy of Ag-RDTs should be evaluated under variable testing conditions i.e. test accuracy using different samples for Ag-RDT, test performed in different days of symptom onset, tests performed in various age groups, etc. The evidence thus generated could be used to evaluate the test accuracy in further detail which could therefore guide clinical use of Ag-RDT as well as direct scientific community to generate testing algorithms for COVID-19 using Ag-RDTs in settings where RT-PCR might not be readily accessible.

## Conclusions

The review on diagnostic accuracy of Ag-RDTs with studies only from low and middle income countries demonstrated pooled sensitivity value approaching the minimum performance requirement for diagnosis of COVID-19 by WHO with specificity value meeting the specified requirement. Ag-RDTs therefore, have the potential to be used as a screening tool for SARS-CoV-2 detection in low resource settings where RT-PCR might not be readily accessible. However, caution should be exercised over interpretation of negative results owing to the lower sensitivity of Ag-RDTs.

## Supporting information

S1 File PRISMA-DTA checklist. (PDF)

S2 File Study protocol registered in PROSPERO. (PDF)

S3 File Search strategy in PubMed. (DOCX)
