# Supplementary material for: Diagnostic accuracy of antigen-detection rapid diagnostic tests for diagnosis of COVID-19 in low-and middle-income countries: A systematic review and meta-analysis
Source: PLOS Glob Public Health. 2022 Apr 11;2(4):e0000358. doi: 10.1371/journal.pgph.0000358 (PMC10021667; doi:10.1371/journal.pgph.0000358)
Supplement: S2 File — (PDF) [file pgph.0000358.s002.pdf]

National Institute for Health Research

UNIVERSITY of York
Centre for Reviews and Dissemination

Systematic review

1. * Review title.

Give the title of the review in English

Effectiveness of antigen-detection rapid diagnostic tests for diagnosis of COVID-19 in low- and middle-income countries: a systematic review and meta-analysis

2. Original language title.

For reviews in languages other than English, give the title in the original language. This will be displayed with the English language title.

3. * Anticipated or actual start date.

Give the date the systematic review started or is expected to start.

20/05/2021

4. * Anticipated completion date.

Give the date by which the review is expected to be completed.

20/05/2022

5. * Stage of review at time of this submission.

Tick the boxes to show which review tasks have been started and which have been completed. Update this field each time any amendments are made to a published record.

Reviews that have started data extraction (at the time of initial submission) are not eligible for inclusion in PROSPERO. If there is later evidence that incorrect status and/or completion date has been supplied, the published PROSPERO record will be marked as retracted.

This field uses answers to initial screening questions. It cannot be edited until after registration.

The review has not yet started: No

Review stage	Started	Completed
Preliminary searches	Yes	Yes
Piloting of the study selection process	Yes	Yes
Formal screening of search results against eligibility criteria	Yes	Yes
Data extraction	Yes	Yes
Risk of bias (quality) assessment	Yes	Yes
Data analysis	Yes	Yes

Provide any other relevant information about the stage of the review here.

6. * Named contact.

The named contact is the guarantor for the accuracy of the information in the register record. This may be any member of the review team.

Sagar Pandey

Email salutation (e.g. "Dr Smith" or "Joanne") for correspondence:

Dr Pandey

7. * Named contact email.

Give the electronic email address of the named contact.

sagarpandey.med@gmail.com

8. Named contact address

Give the full institutional/organisational postal address for the named contact.

Santawan, Dhanewa, Bardaghat Municipality Ward no.13, Lumbini 5, Nepal.

9. Named contact phone number.

Give the telephone number for the named contact, including international dialling code.

+977-9861311580

10. * Organisational affiliation of the review.

Full title of the organisational affiliations for this review and website address if available. This field may be completed as 'None' if the review is not affiliated to any organisation.

B.P.K.I.H.S.

Organisation web address:

11. * Review team members and their organisational affiliations.

Give the personal details and the organisational affiliations of each member of the review team. Affiliation refers to groups or organisations to which review team members belong. **NOTE: email and country now MUST be entered for each person, unless you are amending a published record.**

Dr Sagar Pandey. B.P.K.I.H.S.
Dr Amrit Devkota. B.P.K.I.H.S.
Dr Ujwal Raut. B.P.K.I.H.S.
Dr Anuradha Raut. Nepal Medical College
Dr Arisa Poudel. Institute of Medicine, Tribhuvan University
Dr Dikshya Karki. Kathmandu Medical College
Dr Chet Bahadur Ranabhat. B.P.K.I.H.S.

12. * Funding sources/sponsors.

Details of the individuals, organizations, groups, companies or other legal entities who have funded or sponsored the review.

None

Grant number(s)

State the funder, grant or award number and the date of award

13. * Conflicts of interest.

List actual or perceived conflicts of interest (financial or academic).

None

14. Collaborators.

Give the name and affiliation of any individuals or organisations who are working on the review but who are not listed as review team members. **NOTE: email and country must be completed for each person, unless you are amending a published record.**

Dr Rolina Dhital. Health Action and Research
Dr Richa Shah. Health Action and Research
Dr Carmina Shrestha. Health Action and Research

15. * Review question.

State the review question(s) clearly and precisely. It may be appropriate to break very broad questions down into a series of related more specific questions. Questions may be framed or refined using PI(E)COS or similar where relevant.

What is the effectiveness of antigen-detection rapid diagnostic tests (Ag-RDTs) for diagnosis of COVID-19 in low- and middle-income countries?

16. * Searches.

State the sources that will be searched (e.g. Medline). Give the search dates, and any restrictions (e.g. language or publication date). Do NOT enter the full search strategy (it may be provided as a link or attachment below.)

Sources (database): PubMed, EMBASE, CINAHL, Scopus, Google Scholar.

Search dated: The search window will be limited to published literatures between January 2020 to June 2021.

Language: English only

Searches will be rerun prior to the final analysis.

17. URL to search strategy.

Upload a file with your search strategy, or an example of a search strategy for a specific database, (including the keywords) in pdf or word format. In doing so you are consenting to the file being made publicly accessible. Or provide a URL or link to the strategy. Do NOT provide links to your search **results**.

Alternatively, upload your search strategy to CRD in pdf format. Please note that by doing so you are consenting to the file being made publicly accessible.

Do not make this file publicly available until the review is complete

18. * Condition or domain being studied.

Give a short description of the disease, condition or healthcare domain being studied in your systematic review.

Diagnosis of COVID-19 via Ag-RDTs.

19. * Participants/population.

Specify the participants or populations being studied in the review. The preferred format includes details of both inclusion and exclusion criteria.

The participants will include patients living in low- and middle-income countries suspected of having COVID-19 infection based on typical COVID-19 symptoms or known exposure to COVID-19 or living in an area with community transmission of COVID-19, known COVID-19 infection, and those with known negative COVID-19 infection.

Low- and middle-income countries include low-income countries defined as those with a Gross National Income (GNI) per capita of $1, 035 or less in 2019; lower middle-income countries as those with a GNI per capita between $1, 036 and $4, 045 and upper middle-income countries as those with a GNI per capita between $4, 046 and $12, 535 as per World Bank .

20. * Intervention(s), exposure(s).

Give full and clear descriptions or definitions of the interventions or the exposures to be reviewed. The preferred format includes details of both inclusion and exclusion criteria.

Ag-RDTs are designed to directly detect SARS CoV-2 viral proteins produced by the replicating virus. Most Ag-RDTs for COVID-19 use a sandwich immunodetection method employing a simple-to-use lateral flow test format with techniques such as ELISA, chromogenic based or fluorescence-based detection. In the case of SARS-CoV-2 Ag-RDTs the target analyte is often the virus' nucleocapsid protein, preferred because of its relative abundance.

After collecting the respiratory specimen and applying it to the test strip, results are read by the operator within 10 to 30 minutes with or without the aid of a reader instrument. Most of the currently manufactured

tests require nasal or nasopharyngeal or oropharyngeal swab samples or bronchoalveolar lavage/ endotracheal aspirate. The rapid turnaround time and availability of Ag-RDTs at or near the place where a specimen is collected makes it a good point of care test.

The performance of an Ag-RDTs is determined by the sensitivity and specificity of the test to detect a SARS CoV-2 infection compared with a reference standard, Nucleic Acid Amplification Test, NAAT (generally reverse transcriptase polymerase chain reaction or RT-PCR)

.

RDTs which detect antibodies against COVID-19 will not be included in this study.

21. * Comparator(s)/control.

Where relevant, give details of the alternatives against which the intervention/exposure will be compared (e.g. another intervention or a non-exposed control group). The preferred format includes details of both inclusion and exclusion criteria.

Ag-RDTs will be compared to RT-PCR test as a reference standard.

22. * Types of study to be included.

Give details of the study designs (e.g. RCT) that are eligible for inclusion in the review. The preferred format includes both inclusion and exclusion criteria. If there are no restrictions on the types of study, this should be stated.

Studies evaluating the diagnostic accuracy of Ag-RDTs which meet the criteria for point of care test, against RT-PCR used as a reference standard will be included in our study. Prospective or retrospective cohort studies, cross-sectional studies, case control studies, randomized clinical trials as well as non-randomized experimental studies will be included. We will consider only peer reviewed publications in our study unless there is a paucity of publications meeting all of the inclusion criteria. Studies in which diagnostic accuracy of Ag-RDT was determined in a sample of less than 100 will be excluded so that a more reliable estimate of diagnostic accuracy could be obtained.

23. Context.

Give summary details of the setting or other relevant characteristics, which help define the inclusion or exclusion criteria.

Studies were considered for inclusion if they assessed the diagnostic accuracy of rapid immunoassay for COVID-19 against a reference standard of RT-PCR.

Studies were excluded if the Ag-RDTs itself was used as a reference standard or if only positive or negative Ag-RDTs were confirmed with a reference standard RT-PCR.

Only original studies that described their methods and reported enough data for the construction of the standard two-by-two table were included. Editorials, letters to the editors, and conference abstracts were excluded since they usually contained insufficient information on many important data items relevant to the investigation of sources of heterogeneity (such as patient characteristics, type of specimen, point-of-care use, etc.) and the ascertainment of methodological quality (blind procedures, patient selection, etc.)

24. * Main outcome(s).

Give the pre-specified main (most important) outcomes of the review, including details of how the outcome is defined and measured and when these measurement are made, if these are part of the review inclusion criteria.

The sensitivity and specificity of Ag-RDTs is the primary outcome. Sensitivity is the percentage of cases positive by a Nucleic Acid Amplification Test (NAAT) reference standard that are detected as positive by the Ag-RDTs under evaluation. Specificity is the percentage of cases negative by a NAAT reference standard that are detected as negative by the Ag-RDTs under evaluation.

Studies were considered for inclusion if they assessed the diagnostic accuracy of rapid immunoassay in terms of sensitivity and specificity for COVID-19, against a reference standard of RT-PCR.

Measures of effect

Please specify the effect measure(s) for you main outcome(s) e.g. relative risks, odds ratios, risk difference, and/or 'number needed to treat.

25. * Additional outcome(s).

List the pre-specified additional outcomes of the review, with a similar level of detail to that required for main outcomes. Where there are no additional outcomes please state ‘None’ or ‘Not applicable’ as appropriate to the review

Depending upon the available data, we may also measure positive and negative predictive values of Ag-RDTs as well as area under the curve from a Receiving operating characteristics (ROC) curve.

Measures of effect

Please specify the effect measure(s) for you additional outcome(s) e.g. relative risks, odds ratios, risk difference, and/or 'number needed to treat.

26. * Data extraction (selection and coding).

Describe how studies will be selected for inclusion. State what data will be extracted or obtained. State how this will be done and recorded.

Study selection

Three reviewers SP, AP and AD will independently screen and retrieve the studies using the search strategy and from additional sources. Studies will be further reviewed for eligibility by screening titles followed by abstracts. Full-text articles of narrowed down abstracts will then be assessed for eligibility. Three reviewers AR, UR and DK will check decisions. Upon disagreements, all six reviewers will review the inclusion and exclusion criteria and decision for inclusion will be upon the majority's decision. The principal investigator (SP) will make the final decision after thorough reviewing when an agreement cannot be reached. Zotero, a research tool to collect, organize, and manage research publications will be used to keep a record, and remove duplicates.

Data extraction

All studies retrieved from our search strategies matching our PICO questions and inclusion criteria will be imported to Zotero. Titles of the studies will be first screened followed by abstracts. Screened studies will be placed into appropriate subfolders created in Zotero based on decisions to include or exclude. Full text of all eligible studies will be retrieved and assessed for eligibility. Final data will be extracted from the included studies. The following information will be extracted (if available): demographics details of study participants, total number of study participants, type of intervention, type of specimen used in Ag-RDTs and outcome measures recorded i.e. sensitivity and specificity. All records will be entered into an excel spreadsheet. In case of any missing data, authors of the study will be contacted for additional information. AR, UR and DK will independently extract the data, and SP, AP and AD will check the extracted data. Upon disagreements, all six will review the final extracted data, and disagreements will be resolved through discussion on inclusion and exclusion criteria and via majority's decision.

27. * Risk of bias (quality) assessment.

State which characteristics of the studies will be assessed and/or any formal risk of bias/quality assessment tools that will be used.

All six reviewers will independently assess the risk of bias and the quality of the study. Disagreements between the authors will be resolved by further discussion and consensus between the researchers.

Study tools such as The Quality Assessment of Diagnostic Accuracy Studies (QUADAS)-2 tool will be used to assess the methodological quality of all studies to be included in this systematic review. QUADAS-2 consists of four key domains: patient selection, index test, reference standard, and flow and timing, and we will assess all the domains for risk of bias potential, and the first three domains for applicability concerns. Based on this, risk of bias will be judged as “low,” “high,” or “unclear.

28. * Strategy for data synthesis.

Describe the methods you plan to use to synthesise data. This **must not be generic text** but should be **specific to your review** and describe how the proposed approach will be applied to your data. If meta-analysis is planned, describe the models to be used, methods to explore statistical heterogeneity, and software package to be used.

We will provide a systematic narrative synthesis of the findings from the included studies. Description of each of the included studies will be provided summarizing the features: intervention, population groups, study design, study setting, outcome, bias, and quality of evidence.

Only where appropriate, we will combine data from studies that have used the same type of intervention and outcome measure and perform quantitative analysis of data through metanalysis. We aim to identify the sensitivity, specificity of antigen based rapid diagnostic test for diagnosis of COVID-19. Postitive predictive value, negative value and ROC curve will be constructed as a secondary outcome. We will assess the heterogeneity using I^2 test. We use either fixed effect model or random effects model depending on the heterogeneity of the included studies.

29. * Analysis of subgroups or subsets.

State any planned investigation of ‘subgroups’. Be clear and specific about which type of study or participant will be included in each group or covariate investigated. State the planned analytic approach.

Analysis of subgroups will be performed depending upon Ag-RDTs test brands used, type of specimen, and participant characteristics.

30. * Type and method of review.

Select the type of review, review method and health area from the lists below.

Type of review

Cost effectiveness
No

Diagnostic
Yes

Epidemiologic
No

Individual patient data (IPD) meta-analysis
No

Intervention
No

Living systematic review
No

Meta-analysis

Yes

Methodology
No

Narrative synthesis
No

Network meta-analysis
No

Pre-clinical
No

Prevention
No

Prognostic
No

Prospective meta-analysis (PMA)
No

Review of reviews
No

Service delivery
No

Synthesis of qualitative studies
No

Systematic review
Yes

Other
No

Health area of the review

Alcohol/substance misuse/abuse
No

Blood and immune system
No

Cancer
No

Cardiovascular
No

Care of the elderly
No

Child health
No

Complementary therapies
No

COVID-19
Yes

For COVID-19 registrations please tick all categories that apply. Doing so will enable your record to appear in area-specific searches

Chinese medicine
Diagnosis
Epidemiological
Genetics
Health impacts
Immunity
Long COVID
Mental health
PPE
Prognosis
Public health intervention
Rehabilitation
Service delivery
Transmission
Treatments
Vaccines
Other

Crime and justice
No

Dental
No

Digestive system
No

Ear, nose and throat
No

Education
No

Endocrine and metabolic disorders
No

Eye disorders
No

General interest
No

Genetics
No

Health inequalities/health equity
No

Infections and infestations
No

International development
No

Mental health and behavioural conditions
No

Musculoskeletal

No

Neurological
No

Nursing
No

Obstetrics and gynaecology
No

Oral health
No

Palliative care
No

Perioperative care
No

Physiotherapy
No

Pregnancy and childbirth
No

Public health (including social determinants of health)
No

Rehabilitation
No

Respiratory disorders
No

Service delivery
No

Skin disorders
No

Social care
No

Surgery
No

Tropical Medicine
No

Urological
No

Wounds, injuries and accidents
No

Violence and abuse
No

31. Language.

Select each language individually to add it to the list below, use the bin icon to remove any added in error.
English

There is an English language summary.

32. * Country.

Select the country in which the review is being carried out. For multi-national collaborations select all the countries involved.

Nepal

33. Other registration details.

Name any other organisation where the systematic review title or protocol is registered (e.g. Campbell, or The Joanna Briggs Institute) together with any unique identification number assigned by them. If extracted data will be stored and made available through a repository such as the Systematic Review Data Repository (SRDR), details and a link should be included here. If none, leave blank.

34. Reference and/or URL for published protocol.

If the protocol for this review is published provide details (authors, title and journal details, preferably in Vancouver format)

Add web link to the published protocol.

Or, upload your published protocol here in pdf format. Note that the upload will be publicly accessible.

No I do not make this file publicly available until the review is complete

Please note that the information required in the PROSPERO registration form must be completed in full even if access to a protocol is given.

35. Dissemination plans.

Do you intend to publish the review on completion?

Yes

Give brief details of plans for communicating review findings.?

The research findings will also be shared via publication in a peer reviewed scientific journal.

36. Keywords.

Give words or phrases that best describe the review. Separate keywords with a semicolon or new line. Keywords help PROSPERO users find your review (keywords do not appear in the public record but are included in searches). Be as specific and precise as possible. Avoid acronyms and abbreviations unless these are in wide use.

Antigen-detection; Rapid diagnostic tests; COVID-19; Diagnostic Tests; Point-of-care testing; SARS-CoV-2

37. Details of any existing review of the same topic by the same authors.

If you are registering an update of an existing review give details of the earlier versions and include a full bibliographic reference, if available.

38. * Current review status.

Update review status when the review is completed and when it is published.New registrations must be ongoing so this field is not editable for initial submission.
Please provide anticipated publication date

Review_Completed_not_published

39. Any additional information.

Provide any other information relevant to the registration of this review.

40. Details of final report/publication(s) or preprints if available.

Leave empty until publication details are available OR you have a link to a preprint (NOTE: this field is not editable for initial submission). List authors, title and journal details preferably in Vancouver format.

Give the link to the published review or preprint.
