# Supplementary material for: Diagnostic accuracy of antigen-detection rapid diagnostic tests for diagnosis of COVID-19 in low-and middle-income countries: A systematic review and meta-analysis
Source: PLOS Glob Public Health. 2022 Apr 11;2(4):e0000358. doi: 10.1371/journal.pgph.0000358 (PMC10021667; doi:10.1371/journal.pgph.0000358)
Supplement: S3 File — (DOCX) [file pgph.0000358.s003.docx]

Detailed search strategy for PubMed:

1. (“Afghanistan”[Mesh] OR “Albania”[Mesh] OR “Algeria”[Mesh] OR “American Samoa”[Mesh] OR “Angola”[Mesh] OR “Argentina”[Mesh] OR “Armenia”[Mesh] OR “Azerbaijan”[Mesh] OR “Bangladesh”[Mesh] OR “Republic of Belarus”[Mesh] OR “Belize”[Mesh] OR “Benin”[Mesh] OR “Bhutan”[Mesh] OR “Bolivia”[Mesh] OR “Bosnia and Herzegovina”[Mesh] OR “Botswana”[Mesh] OR “Brazil”[Mesh] OR “Bulgaria”[Mesh] OR “Burkina Faso”[Mesh] OR “Burundi”[Mesh] OR “Cabo Verde”[Mesh] OR “Cambodia”[Mesh] OR “Cameroon”[Mesh] OR “Central African Republic”[Mesh] OR “Chad”[Mesh] OR “China”[Mesh] OR “Colombia”[Mesh] OR “Comoros”[Mesh] OR “Democratic Republic of the Congo”[Mesh] OR “Congo”[Mesh] OR “Costa Rica”[Mesh] OR “Cote d'Ivoire”[Mesh] OR “Cuba”[Mesh] OR “Djibouti”[Mesh] OR “Dominica”[Mesh] OR “Dominican Republic”[Mesh] OR “Ecuador”[Mesh] OR “Egypt”[Mesh] OR “El Salvador”[Mesh] OR “Equatorial Guinea”[Mesh] OR “Eritrea”[Mesh] OR “Eswatini”[Mesh] OR “Ethiopia”[Mesh] OR “Fiji”[Mesh] OR “Gabon”[Mesh] OR “Gambia”[Mesh] OR “Georgia”[Mesh] OR “Ghana”[Mesh] OR “Grenada”[Mesh] OR “Guatemala”[Mesh] OR “Guinea”[Mesh] OR “Guinea-Bissau”[Mesh] OR “Guyana”[Mesh] OR “Haiti”[Mesh] OR “Honduras”[Mesh] OR “India”[Mesh] OR “Indonesia”[Mesh] OR “Iran”[Mesh] OR “Iraq”[Mesh] OR “Jamaica”[Mesh] OR “Jordan”[Mesh] OR “Kazakhstan”[Mesh] OR “Kenya”[Mesh] OR “Democratic People’s Republic of Korea”[Mesh] OR “Kosovo”[Mesh] OR “Kyrgyzstan”[Mesh] OR “Laos”[Mesh] OR “Lebanon”[Mesh] OR “Lesotho”[Mesh] OR “Liberia”[Mesh] OR “Libya”[Mesh] OR “Madagascar”[Mesh] OR “Malawi”[Mesh] OR “Malaysia”[Mesh] OR “Indian Ocean Islands”[Mesh] OR “Mali”[Mesh] OR “Mauritania”[Mesh] OR “Mauritius”[Mesh] OR “Mexico”[Mesh] OR “Micronesia”[Mesh] OR “Moldova”[Mesh] OR “Mongolia”[Mesh] OR “Montenegro”[Mesh] OR “Morocco”[Mesh] OR “Mozambique”[Mesh] OR “Myanmar”[Mesh] OR “Namibia”[Mesh] OR “Nepal”[Mesh] OR “Nicaragua”[Mesh] OR “Niger”[Mesh] OR “Nigeria”[Mesh] OR “Republic of North Macedonia”[Mesh] OR “Pakistan”[Mesh] OR “Papua New Guinea”[Mesh] OR “Paraguay”[Mesh] OR “Peru”[Mesh] OR “Philippines”[Mesh] OR “Romania”[Mesh] OR “Russia”[Mesh] OR “Rwanda”[Mesh] OR “Samoa”[Mesh] OR “Sao Tome and Principe”[Mesh] OR “Senegal”[Mesh] OR “Serbia”[Mesh] OR “Sierra Leone”[Mesh] OR “Melanesia”[Mesh] OR “Somalia”[Mesh] OR “South Africa”[Mesh] OR “South Sudan”[Mesh] OR “Sri Lanka”[Mesh] OR “Saint Lucia”[Mesh] OR “Saint Vincent and the Grenadines”[Mesh] OR “Sudan”[Mesh] OR “Suriname”[Mesh] OR “Syria”[Mesh] OR “Tajikistan”[Mesh] OR “Tanzania”[Mesh] OR “Thailand”[Mesh] OR “Timor-Leste”[Mesh] OR “Togo”[Mesh] OR “Tonga”[Mesh] OR “Tunisia”[Mesh] OR “Turkey”[Mesh] OR “Turkmenistan”[Mesh] OR “Uganda”[Mesh] OR “Ukraine”[Mesh] OR “Uzbekistan”[Mesh] OR “Vanuatu”[Mesh] OR “Venezuela”[Mesh] OR “Vietnam”[Mesh] OR “Yemen”[Mesh] OR “Zambia”[Mesh] OR “Zimbabwe”[Mesh]) OR “Afghanistan”[Title/Abstract] OR “Albania”[Title/Abstract] OR “Algeria”[Title/Abstract] OR “American Samoa”[Title/Abstract] OR “Angola”[Title/Abstract] OR “Argentina”[Title/Abstract] OR “Armenia”[Title/Abstract] OR “Azerbaijan”[Title/Abstract] OR “Bangladesh”[Title/Abstract] OR “Belarus”[Title/Abstract] OR “Belize”[Title/Abstract] OR “Benin”[Title/Abstract] OR “Bhutan”[Title/Abstract] OR “Bolivia”[Title/Abstract] OR “Bosnia and Herzegovina”[Title/Abstract] OR “Botswana”[Title/Abstract] OR “Brazil”[Title/Abstract] OR “Bulgaria”[Title/Abstract] OR “Burkina Faso”[Title/Abstract] OR “Burundi”[Title/Abstract] OR “Cabo Verde”[Title/Abstract] OR “Cambodia”[Title/Abstract] OR “Cameroon”[Title/Abstract] OR “Central African Republic”[Title/Abstract] OR “Chad”[Title/Abstract] OR “China”[Title/Abstract] OR “Colombia”[Title/Abstract] OR “Comoros”[Title/Abstract] OR “Democratic Republic of the Congo”[Title/Abstract] OR “Congo”[Title/Abstract] OR “Costa Rica”[Title/Abstract] OR “Cote d'Ivoire”[Title/Abstract] OR “Côte d'Ivoire”[Title/Abstract] OR “Cuba”[Title/Abstract] OR “Djibouti”[Title/Abstract] OR “Dominica”[Title/Abstract] OR “Dominican Republic”[Title/Abstract] OR “Ecuador”[Title/Abstract] OR “Egypt”[Title/Abstract] OR “El Salvador”[Title/Abstract] OR “Equatorial Guinea”[Title/Abstract] OR “Eritrea”[Title/Abstract] OR “Eswatini”[Title/Abstract] OR “Ethiopia”[Title/Abstract] OR “Fiji”[Title/Abstract] OR “Gabon”[Title/Abstract] OR “Gambia”[Title/Abstract] OR “Georgia”[Title/Abstract] OR “Ghana”[Title/Abstract] OR “Grenada”[Title/Abstract] OR “Guatemala”[Title/Abstract] OR “Guinea”[Title/Abstract] OR “Guinea-Bissau”[Title/Abstract] OR “Guyana”[Title/Abstract] OR “Haiti”[Title/Abstract] OR “Honduras”[Title/Abstract] OR “India”[Title/Abstract] OR “Indonesia”[Title/Abstract] OR “Iran”[Title/Abstract] OR “Iraq”[Title/Abstract] OR “Jamaica”[Title/Abstract] OR “Jordan”[Title/Abstract] OR “Kazakhstan”[Title/Abstract] OR “Kenya”[Title/Abstract] OR “Kiribati”[Title/Abstract] OR “Democratic People’s Republic of Korea”[Title/Abstract] OR “North Korea”[Title/Abstract] OR “Kosovo”[Title/Abstract] OR “Kyrgyzstan”[Title/Abstract] OR “Kyrgyz Republic”[Title/Abstract] OR “Laos”[Title/Abstract] OR “Lao PDR”[Title/Abstract] OR “Lebanon”[Title/Abstract] OR “Lesotho”[Title/Abstract] OR “Liberia”[Title/Abstract] OR “Libya”[Title/Abstract] OR “Madagascar”[Title/Abstract] OR “Malawi”[Title/Abstract] OR “Malaysia”[Title/Abstract] OR “Maldives”[Title/Abstract] OR “Mali”[Title/Abstract] OR “Marshall Islands”[Title/Abstract] OR “Mauritania”[Title/Abstract] OR “Mauritius”[Title/Abstract] OR “Mexico”[Title/Abstract] OR “Federated States of Micronesia”[Title/Abstract] OR “Moldova”[Title/Abstract] OR “Mongolia”[Title/Abstract] OR “Montenegro”[Title/Abstract] OR “Morocco”[Title/Abstract] OR “Mozambique”[Title/Abstract] OR “Myanmar”[Title/Abstract] OR “Namibia”[Title/Abstract] OR “Nauru”[Title/Abstract] OR “Nepal”[Title/Abstract] OR “Nicaragua”[Title/Abstract] OR “Niger”[Title/Abstract] OR “Nigeria”[Title/Abstract] OR “North Macedonia”[Title/Abstract] OR “Pakistan”[Title/Abstract] OR “Papua New Guinea”[Title/Abstract] OR “Paraguay”[Title/Abstract] OR “Peru”[Title/Abstract] OR “Philippines”[Title/Abstract] OR “Romania”[Title/Abstract] OR “Russia”[Title/Abstract] OR “Russian Federation”[Title/Abstract] OR “Rwanda”[Title/Abstract] OR “Samoa”[Title/Abstract] OR “Sao Tome and Principe”[Title/Abstract] OR “São Tomé and Principe”[Title/Abstract] OR “Senegal”[Title/Abstract] OR “Serbia”[Title/Abstract] OR “Sierra Leone”[Title/Abstract] OR “Solomon Islands”[Title/Abstract] OR “Somalia”[Title/Abstract] OR “South Africa”[Title/Abstract] OR “South Sudan”[Title/Abstract] OR “Sri Lanka”[Title/Abstract] OR “Saint Lucia”[Title/Abstract] OR “Saint Vincent and the Grenadines”[Title/Abstract] OR “Sudan”[Title/Abstract] OR “Suriname”[Title/Abstract] OR “Syria”[Title/Abstract] OR “Syrian Arab Republic”[Title/Abstract] OR “Tajikistan”[Title/Abstract] OR “Tanzania”[Title/Abstract] OR “Thailand”[Title/Abstract] OR “Timor-Leste”[Title/Abstract] OR “Togo”[Title/Abstract] OR “Tonga”[Title/Abstract] OR “Tunisia”[Title/Abstract] OR “Turkey”[Title/Abstract] OR “Turkmenistan”[Title/Abstract] OR “Tuvalu”[Title/Abstract] OR “Uganda”[Title/Abstract] OR “Ukraine”[Title/Abstract] OR “Uzbekistan”[Title/Abstract] OR “Vanuatu”[Title/Abstract] OR “Venezuela”[Title/Abstract] OR “Vietnam”[Title/Abstract] OR “West Bank and Gaza”[Title/Abstract] OR “Yemen”[Title/Abstract] OR “Zambia”[Title/Abstract] OR “Zimbabwe”[Title/Abstract] NOT (“Seychelles”[Mesh] OR “Seychelles”[Title/Abstract])

#2: "covid-19 testing"[MeSH Terms] OR "Antigen based rapid diagnostic test"[All Fields] OR "diagnostic test" OR "lateral flow antigen" OR "lateral flow antigen detection" OR "lateral flow assa*" OR "Point of care testing"[Mesh]

#3: "coronavirus infections"[MeSH Terms] OR "COVID-19"[MeSH Terms] OR "coronavirus disease 2019"[All Fields] OR "COVID19"[All Fields] OR "SARS-CoV-2"[All Fields]

Final Search: #1 AND #2 AND #2

Filters: 2020 and 2021 publication year

Search Results: 1240 articles
